# Hydroxyethylcellulose as a methotrexate carrier in anticancer therapy

**DOI:** 10.1007/s10637-020-00972-9

**Published:** 2020-07-08

**Authors:** Jarosław Ciekot, Mateusz Psurski, Katarzyna Jurec, Janusz Boratyński

**Affiliations:** Department of Experimental Oncology, Hirszfeld Institute of Immunology and Experimental Therapy, Polish Academy of Sciences, 53-114 Wroclaw, Poland

**Keywords:** Hydroxyethylcellulose, Methotrexate, Drug-carrier conjugate, Anticancer therapy

## Abstract

Clinical and experimental cancer therapy is multifaceted; one such facet is the use of drug carriers. Drug carriers are various nano- and macromolecules, e.g., oligosaccharides, proteins, and liposomes. The present study aimed to verify the suitability of cellulose as a carrier for methotrexate (MTX). Hydroxyethylcellulose, with a molecular weight of 90 kDa and soluble in water, was used. Methotrexate was linked to cellulose by methyl ester bonds. A conjugate containing on average 9.5 molecules of MTX per molecule of cellulose was developed. Gel filtration HPLC analysis showed that the conjugate contained approximately 2% free drug. Dynamic light scattering analysis showed an increase in the polydispersity of the conjugate. The degradation of the conjugate in phosphate buffer and plasma followed first-order kinetics. The conjugate showed the lowest stability (half-life 154 h) in plasma. The conjugate showed 10-fold lower cytotoxicity to the 4 T1 mammary tumour cell line than the free drug. In the in vivo experiment to treat orthotopically implanted mammary tumours, the conjugate and the free drug, both applied intravenously, showed maximum inhibition of tumour growth of 48.4% and 11.2%, respectively. In conclusion, cellulose, which is a non-biodegradable chain glucose polymer, can be successfully used as a drug carrier, which opens up new research perspectives.

## Introduction

Drug carrier systems have been developed for approximately 70 years, although this idea was proposed more than 100 years ago by Ehrlich [1]. These systems are multifaceted issues that include, inter alia, pharmacology, protein research, and chemical modifications of macromolecules. From a therapeutic point of view, the coupling of low-molecular-weight drugs with a carrier offers many advantages. The most important benefits include minimizing the toxic effects and immunogenicity of the drug [2], controlled drug release in the plasma or at the target site [3], and extensive modulation of drug metabolism and its interaction with multidrug resistance proteins [4]. The most important advantage of conjugates is the extension of the drug’s circulation time within the body. Systems with a molecular weight above 50 kDa remain in the body for a much longer time because of slowed plasma clearance and improved accumulation at the tumour site [5]. The negative charge on the polymer surface further reduces the uptake resulting from a first-pass effect. The reason for this reduction is that the vascular endothelial surfaces are coated with negatively charged components such as chondroitin sulfate, heparan sulfate, and glycocalyx. The use of a drug delivery system with a negative charge on the surface extends the drug’s half-life in the body [6]. Despite extensive research, only a few systems have found applications in therapy, for example, PEGylated liposomes containing doxorubicin (Doxil) [7]. Drug-carrier conjugates, despite many theoretical advantages, have just begun to play a relevant role, as exemplified by the introduction to the market of the Adcetris conjugate by Seattle Genetics in 2011 [8]. The Adcetris conjugate is the first monoclonal antibody, of which over 70 have been used in medicine [9, 10], conjugated with a cancer drug that is approved for clinical use.

Oligosaccharides, such as dextran and hydroxyethyl starch, play a significant role in investigations of drug-carrier systems in the field of experimental oncology [11, 12]. Oligosaccharide carriers have several advantageous features. In practical medicine, they have been used in plasma substitute preparations; they also enable the binding of therapeutic substances through hydroxyl groups or generated aldehyde groups in chemically modified versions [13]. Cellulose, unlike the abovementioned polymers, is not biodegradable in the human body because of the presence of β-1,4-glycosidic bonds between glucose subunits. This feature can determine the beneficial and unfavourable properties of cellulose-based conjugates, and therefore, the results of studies based on this natural polymer need to be verified. Other non-biodegradable drug carriers used in studies are linear (unbranched) polymers—*N*-(2-hydroxypropyl)methacrylamide [7]—or dendrimers characterized by a spherical structure [14].

In a hybrid molecule, it is extremely difficult to assess which element, namely, the drug, the carrier, or the type of bond, plays a decisive role in the biological activity of the preparation. The carrier, as an important component, is responsible for the tropic, physicochemical, and biological properties of the substance bound with it. The development of conjugate research has led researchers to pay attention to the geometry of molecules that are potential candidates for a drug carrier. Studies dedicated to this topic have shown that elongated or filamentous nanoparticles, which include cellulose derivatives, have clear advantages over spherical nanoparticles, considering the ratio of surface to volume, the rate of removal from the body, and the mechanism of elimination [15]. The drug carrier system 2-hydroxyethylcellulose (HEC) possesses three hydroxyl groups available for modification on the surface of each monomer glucose, which allow the carrier to be coupled with active compounds containing *fluorophore* groups (for imaging) and compounds that afford tropicity to drug-carrier systems.

Cellulose is a nontoxic, branched-chain polymer that is the dominant biomass component in nature [16]. One of its many derivatives is HEC, which exhibits excellent properties that make it a suitable candidate drug carrier. These properties include its molecular weight (approximately 90 kDa), neutral charge, and water solubility. A wide panel of HEC preparations is available because of the use of this polymer in industry; it is also considered an excellent thickening and stabilizing agent due to its chemical stability and biocompatibility [17]. The expected properties of both the carrier and the drug must be maintained in conjugates. Given the above advantages of HEC, it is reasonable to test the suitability of this polymer as a carrier for methotrexate (MTX), which inhibits dihydrofolate reductase, thymidylate synthase, 5-aminoimidazole-4-carboxamide ribonucleotide transformylase, and trifunctional purine biosynthetic protein (GART) [18, 19]. This study aimed to evaluate the potential value of high-molecular-weight HEC conjugates with MTX and to assess their physicochemical properties and biological activity.

## Materials and methods

### Synthesis of conjugate

Methotrexate was activated using N,N′-dicyclohexylcarbodiimide following the previously described method introduced in our laboratory [12]. Five HEC conjugates with MTX with various degrees of substitution were synthesized. The first step in the synthetic procedure was the dissolution of 1 g of HEC (Sigma-Aldrich) in 30 ml of 0.05 M sodium carbonate. The second step was the addition of various amounts of MTX anhydride solution (concentration of 100 mg/ml) at a rate of 1 ml/min, with vigorous stirring, maintaining the pH above 10.5 (using titration with 1 M NaOH). The molar ratios of the reactants was of 20 to 100 MTX molecules per HEC molecule. The reaction product was a hybrid molecule containing MTX bound with HEC and water as a byproduct. After adding the entire amount of MTX, the mixture was neutralized to a pH of 7 by using 10% acetic acid. The free drug was removed by dialysis using a Pellicon® XL tangential-flow filter (regenerated cellulose membrane, 10 kDa cutoff, 50 cm^2^ filtration area) at a flow rate of 15 ml/min. In the first stage, the conjugate was dialyzed to 0.1 M sodium bicarbonate until a fixed level of conjugate purification from the free drug (below 5%) was obtained. In the second stage, the preparation was dialyzed against Milli-Q water (resistivity: 18.2 MΩ·cm).

### Determination of conjugate substitution degree

The total content of the drug in the preparations was determined spectrophotometrically. The absorbance was measured by a validated method with 0.1 M sodium bicarbonate solution at 372 nm. All measurements were performed at room temperature using a Specord 250 spectrophotometer in 1 cm cuvettes [20].

The free drug content in the preparations was determined by gel filtration on a Superdex® 30 column (34 μm, 4.6 × 150 mm) (GE Healthcare, Little Chalfont, UK). The measurements were performed using a Dionex Ultimate 3000 chromatograph equipped with an LPG-3400SD pump, a WPS-3000 T(B) FC analytical autosampler, a TCC-3000SD column oven, and a DAD-3000 diode array detector. Isocratic elution with 0.1 M sodium bicarbonate at a flow rate of 0.4 ml/min was used. The injection volume was 10 μl. Detection was performed at a wavelength of 302 nm because of the higher molar absorption of MTX [20]. To determine the content of HEC, it was necessary to hydrolyse the conjugate in 10 mM sodium hydroxide for 24 h at room temperature. The chromatographic separation was carried out on a Superdex® 30 column (34 μm, 4.6 × 150 mm) (GE Healthcare). Isocratic elution with 0.1 M sodium bicarbonate at a flow of 0.4 ml/min was then performed. The injection volume was 10 μl. Detection was carried out at a temperature of 35 °C and a detector sensitivity of 512×. Calibration was performed by injecting HEC at concentrations ranging from 5.556 to 55.56 μM. The dependence of the surface area under the peak on the concentration [μM] is described by the equation *y* = 0.2734*x* − 0.3989. The measurements were performed using a Dionex Ultimate 3000 chromatograph equipped with an LPG-3400SD pump, a WPS-3000 T(B) FC analytical autosampler, a TCC-3000SD column oven, and a Shodex RI 102 refractometer.

### Stability of conjugates

The most active conjugate, HEC-MTX3, was selected for stability studies. The conjugate was diluted with mouse plasma or phosphate buffer at pH 7.2 to a concentration of 200.6 μM based on MTX and incubated at 37 °C. At certain time points, the concentration of HEC-MTX released from the conjugate was measured by gel filtration. The chromatographic separation was performed using a Superdex® 30 column (34 μm, 4.6 × 300 mm) (GE Healthcare). Isocratic elution with 0.1 M sodium bicarbonate at a flow of 0.4 ml/min was used. The injection volume was 10 μl. Detection was performed at a wavelength of 372 nm because of the presence of plasma components. Calibration was performed by injecting MTX at concentrations from 4.013 to 200.6 μM. The dependence of the area under the peak on the concentration [μM] is described by the equation *y* = 0.1852*x* + 0.0038. The measurements were performed using a Dionex Ultimate 3000 chromatograph equipped with an LPG-3400SD pump, a WPS-3000 T(B) FC analytical autosampler, a TCC-3000SD column oven, and a DAD-3000 diode array detector. When calculating the reaction rate constant and the half-life, it was assumed that the release of MTX from the conjugate proceeded according to first-order kinetics.

### Analysis of hydrodynamic parameters

Measurements of hydrodynamic diameter were performed on a Malvern Zetasizer Nano apparatus with backward diffusion of laser light at a wavelength of 633 nm (173°) in quartz cuvettes at 25 °C. Before the measurement, the conjugate was diluted with 10 mM phosphate buffer at pH 7.2, resulting in a final carrier concentration in the test sample of 11.11 μM. The final result of the measurements was obtained by averaging 6–10 independent measurements. The applied coefficient of analyte refraction was 1.520. Buffer viscosity was calculated using Zetasizer 7.11 software (the value was 1.051 cP). Data from hydrodynamic diameter measurements were also analysed using Zetasizer 7.11 software.

Furthermore, measurements of zeta potential were performed on a Malvern Zetasizer Nano apparatus with backward diffusion of laser light at a wavelength of 633 nm (173°) in zeta potential-measuring cuvettes (ZEN1010, Malvern). Before the measurement, the conjugate was diluted with Milli-Q water to obtain a final carrier concentration in the test sample of 11.11 μM. The final result of the measurements was obtained by averaging 10–20 independent measurements. Data from zeta potential measurements were analysed using Zetasizer 7.11 software.

### In vitro *analysis*

An antiproliferative activity assessment was conducted using a standard MTT (3-(4,5-dimethylthiazolyl-2)-2,5-diphenyltetrazolium bromide) assay or SRB (sulforhodamine B) assay [21]. A human biphenotypic B myelomonocytic leukaemia cell line (MV-4-11) and a mouse breast tumour cell line (4 T1) were obtained from American Type Culture Collection (ATCC, Rockville, USA). MV-4-11 cells and 4 T1 cells were cultured in 96-well plates (Sarstedt, Nümbrecht, Germany) at a density of 1 × 10^4^ and 1 × 10^3^ cells per well, respectively. After 24 h of incubation, the cells were exposed to the test preparations (concentrations of HEC-MTX conjugates and MTX were in the range of 0.2–200.6 nM based on the concentration of MTX in the preparation, and the concentration of the HEC carrier control was in the range of 0.02–20.6 nM [analogous to the carrier concentration in the HEC-MTX3 conjugate]). After 72 h of incubation, the MTT test was carried out on the MV-4-11 cell line and the SRB test on 4 T1 cells; the absorbance readings were taken at 570 and 540 nm, respectively, in the Synergy H4 plate reader. The obtained results are presented as IC_50_ (half maximal inhibitory concentration) with its standard deviation, calculated based on Cheburator [22]. The tests were repeated in triplicate.

### In vivo *analysis*

The anticancer activity of the preparations was tested in BALB/cmdb mice (females, Center for Experimental Medicine, University of Bialystok). The 4 T1 cells derived from in vitro culture were implanted orthotopically in the right third nipples of each mouse at a density of 3 × 10^5^ cells/mouse in a volume of 0.05 ml of Hanks’ fluid. The preparations were administered once intravenously to the tail vein at a dose of 40 μmol/kg body weight based on MTX, when the average tumour volume was approximately 100 mm^3^. Groups were marked according to the scheme shown in Table 1.

**Table 1:** Nomenclature of the groups in an in vivo experiment

Name of the group	Description
Control	0.15 M sodium chloride
HEC-MTX3	Conjugate with substitution degree 8.3 (MTX dose 40 μmol/kg body weight)
HEC-MTX5	Conjugate with substitution degree 14.3 (MTX dose 40 μmol/kg body weight)
MTX	Methotrexate (MTX dose 40 μmol/kg body weight)
HEC	Hydroxyethylcellulose (carrier dose equivalent to in the HEC-MTX3 conjugate)
MIX	Mixture of the ingredients tested (MTX dose 40 μmol/kg body weight, carrier dose equivalent to in the HEC-MTX3 conjugate)

Measurements of subcutaneous tumour size and animal weight were performed during the experiment (thrice a week). Tumour volume was calculated according to the equation $TV\left[ mm3\right]=\frac{a^2\ast b}{2}$, where *a* and *b* are the shortest and longest tumour diameters, respectively. Tumour growth inhibition (TGI) was calculated according to the equation $TGI\left[\%\right]=\frac{TV_T}{TV_C}\ast 100-100$, where *TV*_*t*_ refers to the mean tumour volume in the treated group and *TV*_*c*_ refers to the mean tumour volume in the control group.

Statistical analysis was performed for tumours measured on all measurement days. The data were analysed with the Kruskal-Wallis ANOVA nonparametric test. The Kruskal-Wallis ANOVA test was carried out with testing for multiple data comparisons (multiple comparison *p* values [2-tailed]). Differences between particular groups were considered statistically significant when the *p* value was below 0.05.

## Results

HEC conjugates with MTX were obtained in the study through the reaction between the hydroxyl groups of the carrier and MTX anhydride. The resulting conjugate was purified from the free drug to less than 2% free drug. Data from the stoichiometric analysis are presented in Table 2.

**Table 2:** Characteristics of conjugates

Conjugate	C_HEC_ in the synthesis procedure ×10^−3^ [mol]	C_MTX_ in the synthesis procedure ×10^−3^ [mol]	C_HEC_ ×10^−3^ [M]	C_MTX_^all^ ×10^−3^ [M]	MTX-(COOH)_2_ [%]	C_MTX_^bound^ × 10^−3^ [M]	SL
HEC-MTX1	11.11	220.1	0.7271	2.223	1.18	2.196	3.021
HEC-MTX2	11.11	440.1	0.8222	4.445	0.23	4.435	5.394
HEC-MTX3	11.11	660.2	0.5331	4.423	0.19	4.415	8.281
HEC-MTX4	11.11	880.2	0.3757	4.423	0.33	4.408	11.73
HEC-MTX5	11.11	1100	0.3123	4.489	0.37	4.472	14.32

HEC-MTX. C_HEC_—concentration of HEC; C_MTX_^all^—total concentration of MTX; MTX-(COOH)_2_—percentage content of MTX not bound with the carrier; C_MTX_^bound^—concentration of MTX bound with HEC; SL—degree of carrier substitution with the drug [mol MTX/mol HEC].

**Fig. 1:**
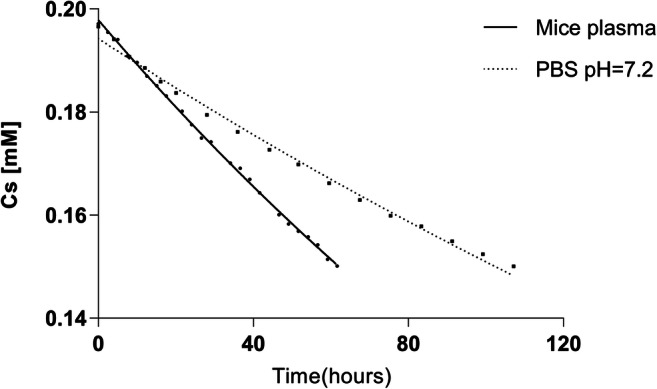
The stability curve for conjugate hydrolysis (first-order kinetics). Concentration of conjugate: 0.2006 mM (based on MTX); *C*_s_—concentration of MTX bound with carrier; solvents—10 mM phosphate buffer at pH 7.2 with ionic strength of 154 mM and mouse plasma

The HEC-MTX3 conjugate decomposed according to first-order kinetics. The conjugate decomposed almost twice as fast in human plasma as in 10 mM phosphate buffer at pH 7.20. This indicates the catalytic effect of plasma components on the decomposition of the conjugate. The stability results of the conjugate are shown in Table 3 and Fig. 1.

**Table 3:** Stability of the HEC-MTX3 conjugate in 10 mM phosphate buffer at pH 7.20 with an ionic strength of 154 mM (PBS) and in mouse plasma

Solution used for incubation	pH	*t*_1/2_ [hours]
10 mM PBS	7.20 ± 0.05	292.5 ± 8.2
Mouse plasma	7.40 ± 0.05	154.7 ± 2.7

*t*_1/2_—half-life equals the time after which half of the drug bound in the conjugate is released.

**Fig. 2:**
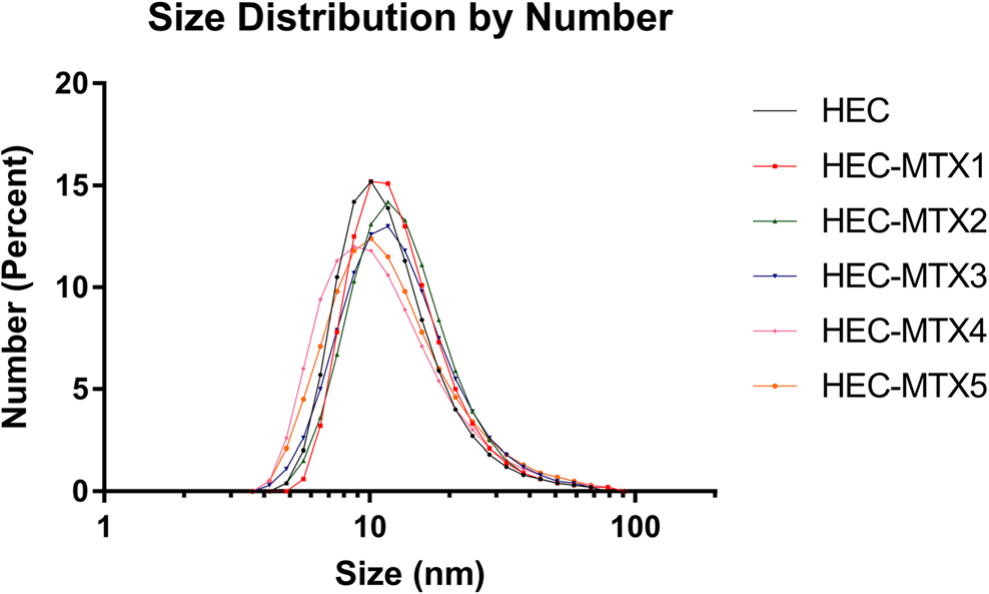
Distribution of the hydrodynamic diameter of the HEC-MTX conjugates in comparison to the HEC carrier. Concentration of conjugate and carrier: 11.11 μM (based on HEC), solvent—10 mM phosphate buffer pH 7.2 with ionic strength of 154 mM

An analysis of hydrodynamic parameters provides information about the hydrodynamic diameter and the zeta potential of the obtained preparations. The hydrodynamic diameter of the free carrier was 9.238 nm. The hydrodynamic diameter of the molecules in the HEC-MTX conjugates with a degree of substitution between 3 and 8 was found to be higher than that of molecules in the carrier, but the hydrodynamic diameter of the molecules in the HEC-MTX conjugates with a degree of substitution between 11 and 14 was found to be lower than that of molecules in the carrier. However, the differences in dynamic diameter between the preparations were very small. The relevant data are shown in Table 4 and Fig. 2. The polydispersity of the conjugates was higher than that observed for the unsubstituted carrier. A negative charge is imparted to the conjugate as a result of the attachment of a drug molecule to an electrically neutral polymer (HEC). The zeta potential of the conjugates decreased as the level of substitution increased. The correlation between the degree of substitution and the zeta potential was described by the equation *y* =  − 0.85 × *x* − 2.286. The determinant coefficient for the equation was found to be 0.981.

**Table 4:** Hydrodynamic parameters of the HEC-MTX conjugates

Conjugate	SL	*D*_H_ [nm]	PDI	ZP [mV]
HEC-MTX1	3.021	10.22 ± 3.311	0.105	−3.1
HEC-MTX2	5.394	10.24 ± 3.678	0.116	−4.22
HEC-MTX3	8.281	9.957 ± 3.745	0.129	−4.62
HEC-MTX4	11.73	8.554 ± 3.373	0.129	−5.56
HEC-MTX5	14.32	8.773 ± 3.321	0.141	−6.68
HEC	–	9.238	0.107	–

SL—degree of carrier substitution with the drug [mol MTX/mol HEC]; *D*_H_—hydrodynamic diameter; PDI—degree of polydispersity; ZP—zeta potential. Concentration of conjugate: 11.11 μM (calculated on HEC), and solvent—10 mM phosphate buffer pH 7.2 with 154 mM ionic strength.

The antiproliferative activity of the preparation was tested in human biphenotypic B myelomonocytic leukaemia (MV-4-11) and murine mammary gland cancer cells (4 T1). The IC_50_ values determined for conjugates and free drug are listed in Table 5. The HEC-MTX preparations showed approximately 3- to 5-fold lower antiproliferative activity on the 4 T1 cell line than free MTX did. The unmodified polymer at a concentration equivalent to the carrier concentration in the HEC-MTX3 conjugate was not cytotoxic to the tumour cell lines tested. The HEC-MTX preparation showed approximately 10- to 12-fold lower antiproliferative activity on the MV-4-11 cell line than free MTX did. The unmodified polymer at a concentration equivalent to the carrier concentration in the HEC-MTX3 conjugate was not cytotoxic to the tested tumour cell lines.

**Table 5:** IC_50_ values for the tested preparations with respect to the MV-4-11 cell line (human biphenotypic B myelomonocytic leukaemia) and 4 T1 cell line (mouse breast tumour)

		MTT		SRB	
Conjugate	SL	MV-4-11		4 T1	
		IC_50_ ± SD (*n* = 4) μg/mL		IC_50_ ± SD (*n* = 4) μg/mL	
HEC-MTX1	3.021	0.776	± 0.256	0.394	± 0.241
HEC-MTX2	5.394	0.694	± 0.247	0.592	± 0.266
HEC-MTX3	8.281	0.743	± 0.325	0.36	± 0.108
HEC-MTX4	11.73	0.793	± 0.301	0.436	± 0.098
HEC-MTX5	14.32	0.857	± 0.248	0.452	± 0.254
MTX		0.069	± 0.025	0.109	± 0.039

HEC-MTX1 to −5—HEC-MTX conjugates with different substitution degrees; MTX—reference compound MTX. All preparations were tested in concentrations in the range of 0.2 to 200.6 nM (MTX concentration in the preparation).

**Fig. 3:**
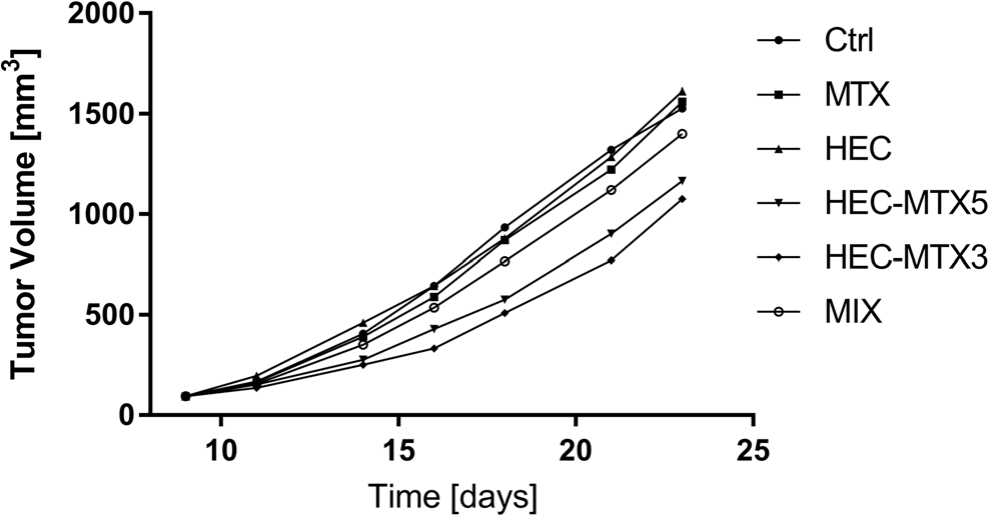
Kinetics of tumour growth in the 4 T1 mouse breast tumour cell line. Mice were subjected to therapy with HEC-MTX3 and HEC-MTX5 conjugates (dose 40 μmol/kg). MTX—positive control MTX; HEC—carrier control; MIX—control of HEC and MTX mixture (molar ratio as in conjugate HEC-MTX3)

**Fig. 4:**
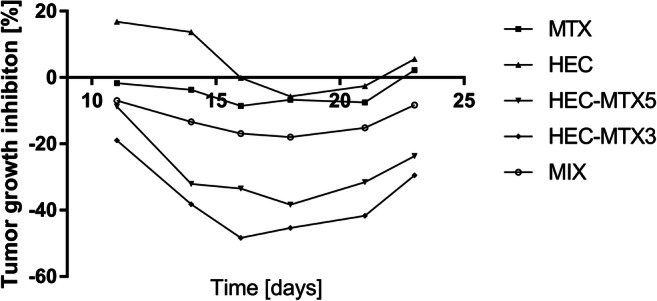
4 T1 tumour growth inhibition (TGI) (Experiment 1). Mice were subjected to therapy with HEC-MTX3 and HEC-MTX5 conjugates (dose 40 μmol/kg). MTX—positive control MTX; HEC—carrier control; MIX—control of HEC and MTX mixture (molar ratio as in conjugate HEC-MTX3)

The in vivo anticancer activity of the HEC-MTX3 and HEC-MTX5 conjugates was determined based on the tumour volume. The percent inhibition of tumour growth is shown in Fig. 3. The toxicity of the preparation was evaluated by monitoring the change in the weight of mice during the experiment. The value of the TGI parameter for the HEC-MTX3 conjugate ranged from 19% on the 11th day of the experiment to 48.4% on the 16th day of the experiment. At the end of the experiment, its TGI value was 29.5%. The value of the TGI parameter for the HEC-MTX5 conjugate was 8.8% on the 11th day of the experiment to 38.4% on the 18th day of the experiment. At the end of the experiment, its TGI value was 23.7% (Fig. 4). The statistical significance of the results is shown in Table 6. For other groups, the TGI parameter values were as follows:

- MTX—TGI did not exceed 8.6%,
- HEC—TGI did not exceed 5.7%,
- MIX—TGI did not exceed 18%.

**Table 6:** The statistical significance of the results

Conjugate	Reference group	*p* < 0,05 Kruskal-Wallis ANOVA nonparametric test
HEC-MTX3	Control	Day:14–23
	MTX	
	MIX	Day:16–23
	HEC	Day:14–23
HEC-MTX5	Control	Day:14–21
	MTX	Day: 18
	MIX	Day:18
	HEC	Day:14–23

Confirming our assumptions, the conjugate did not show toxic effects during the experiment.

Mice were subjected to therapy with the HEC-MTX3 or HEC-MTX5 conjugate (dose 40 μmol/kg). MTX—positive control MTX; HEC—carrier control; MIX—control of HEC and MTX mixture (molar ratio as in conjugate HEC-MTX3).

## Discussion

Studies on drug carrier systems have created limitless opportunities for discovery, generating the need to do research that draws on various fields, including inter alia, drug chemistry, carrier chemistry, coupling agent chemistry, and advanced analytics. The elements of a drug structure that do not affect its activity are predisposed to coupling. MTX, which is one of the oldest anticancer drugs that is still widely used in oncology, was used in this study as the active compound [23]. For MTX and folic acid derivatives, the use of a carboxyl moiety in the coupling does not lead to a loss of biological activity, and the usage of its carboxyl groups enables an efficient synthesis of the conjugates with cellulose, dextran, albumin, etc. The obtained preparations with these carriers have shown higher antitumour activity than the free drug [11, 24–26]. The preparation of active esters or anhydride forms of the drug is a simple and convenient method of obtaining biologically active conjugates with proteins and polysaccharides [11, 25]. The conjugates in this study were obtained from the direct reaction between HEC and MTX anhydride in a molar ratio of 20 to 100 MTX molecules per HEC molecule. This approach ensured the repeatability of the conjugates obtained.

The ideal features of the hybrid nanoparticle include selectivity in recognizing target sites and meeting certain biological criteria (mainly lack of toxicity and immunogenicity), ease of drug binding, and appropriate molar weight of the polymer so that the conjugate circulates in the body long enough and undergoes controlled elimination (it is assumed that the optimal molar weight ranges from 30 to 100 kDa). On the one hand, stability is required, while on the other hand, easy release of the drug may be an advantageous feature in some cases [27–29]. HEC as the carrier fulfils most of the features of an ideal drug carrier. The only parameter not fulfilled is biodegradability in the human body. In our studies, the preparations showed no toxicity resulting from the lack of biodegradability, and additionally, the attachment of MTX to HEC caused the appearance of an additional carboxyl functional group on the polymer surface. An analogous situation to a lack of biodegradability occurs for dendrimers, which are also used in research on drug-carrier systems. In addition, modification of non-biodegradable polymers with neutral or anionic functional groups reduces toxicity compared to cationic functional group modification [30].

The advantage of HEC as a carrier is the lack of toxic effects after a single administration. In the presented approach, a simple method of conjugate synthesis was used, where an integral part of MTX—glutamic acid—was used for coupling. This method of synthesis avoids the use of complicated linkers between the drug and the carrier, and as a result of hydrolysis, molecules of only MTX and HEC were obtained. The developed synthesis method allowed conjugates with varying degrees of substitution to be obtained. In the present study, conjugates with a substitution degree of 3–14 MTX molecules per carrier molecule were obtained. In addition, despite MTX attachment to the carrier, the DLS analysis did not show any significant changes in the hydrodynamic diameter of the conjugates. The size of the unsubstituted polymer and conjugate predisposes it to accumulation in tumours using the enhanced permeability and retention effect (EPR) [31, 32]. An electrically neutral polymer such as HEC becomes negatively charged after MTX binding. Compared to the HES-MTX conjugate, which has a zeta potential of −27.7 mV, the zeta potential of the HEC-MTX conjugates is −3.1 to −6.7 mV [11]. However, even this relatively low zeta potential contributes to reducing nonspecific ionic interactions with the negatively charged cells of the body [30]. Another important parameter determining the usefulness of conjugates in therapy is their stability in plasma. HEC-MTX3 conjugates have a half-life of approximately 150 h in mouse plasma and 300 h in phosphate-buffered saline (PBS) pH = 7.2. For comparison, data on the stability of the commercially used Doxilu formulation (doxorubicin encapsulated in pegylated liposomes) in human plasma and PBS were used. During a 50-h incubation in human plasma, researchers have observed approximately 20% drug release from the formulation, while during an 80-h incubation in PBS pH = 7.5, approximately 15% drug release from the formulation was observed [33]. In this study, 20% of the drug was released from the HEC-MTX conjugate during 50 h of incubation in mouse plasma, and the situation is similar during 80 h of incubation in PBS pH = 7.2, where 20% of the drug was also released. By comparing the results obtained for HEC-MTX conjugates and the literature data for Doxil, we found that the stability of the obtained conjugates is sufficient for clinical applications. The physicochemical parameters of the conjugates described above may result in prolongation of the time of possible accumulation in the tumour with the use of the EPR effect and an increase in the effectiveness of its action at this site [34].

The synthesized formulations’ anti-proliferative activity against 4 T1 tumour cell lines was measured, and the IC_50_ parameter was several times higher for HEC-MTX conjugates than for the free drug. In contrast, the unmodified polymer at a concentration equivalent to the carrier concentration in the HEC-MTX3 conjugate was not cytotoxic to the cancer cell lines. The data obtained represent a property of carrier drug conjugates; the conjugate activity relative to free MTX is usually several times lower. Many researchers have observed that conjugates have lower antiproliferative activity than free drugs, for example, conjugates of dextran with MTX and conjugates of fibrinogen with MTX [12, 25, 35–38]. The full usefulness of conjugates is confirmed only by animal experiments. Compared to other conjugates with MTX as an active substance, the HEC-MTX conjugate showed satisfactory anticancer activity. The anticancer activity of the conjugate can be compared to other published results. The hydroxyethyl starch conjugate with MTX, whose activity was evaluated on the MV-4-11 tumour cell line, showed inhibition of tumour growth at a level of 90% [11]. Conjugates of MTX with albumin showed a beneficial effect on the inhibition of tumour growth. The effect was compounded by using luteinizing hormone-releasing hormone (LHRH) or biotin as an element to afford tropicity. The prolongation of the lifetime of both the albumin-MTX-LHRH conjugate and albumin-MTX-biotin conjugate was approximately 250% [39, 40].

The conjugate presented in this study is one of the few examples of cellulose derivatives used as a drug carrier in intravenous administration. A formulation containing carboxymethylcellulose as a drug delivery system for docetaxel has shown good therapeutic effectiveness [41]. Cellulose derivatives as drug carriers have both disadvantages and advantages. An important feature of cellulose is its stability in biological systems. This feature involves polymer removal after drug dissociation. Hydroxyethylcellulose has a particle width of 3–5 nm and a length of 10–30 nm [42, 43]. The cylindrical shape and size of the carrier allow it to be eliminated by the kidneys for excretion in urine. The pore diameter of the kidney glomerulus is approximately 5 nm, which allows the removal of the linear HEC polymer [44, 45].

In the proposed approach, the active substance is MTX, whose activity goes beyond cancer disease therapy. Accordingly, conjugates of this drug can be used beyond cancer therapy, e.g., in the therapy of autoimmune diseases such as rheumatoid arthritis [46].

## Conclusion

The present data confirm the high antitumour activity of the proposed preparations in comparison to the free drug. Consequently, conjugates using HEC as a drug carrier appear to be a new avenue in the field of drug delivery systems. Thus, conjugates based on cellulose derivatives can be used as carriers of therapeutic substances for phagocytic cells with intracellular pathogens.
